# 3-Amino-5,6-dimethyl-1,2,4-triazin-2-ium nitrate

**DOI:** 10.1107/S1600536813012026

**Published:** 2013-05-11

**Authors:** Souhir Bel Haj Salah, Mohamed Lahbib Mrad, Valeria Ferretti, Frederic Lefebvre, Cherif Ben Nasr

**Affiliations:** a Laboratoire de Chimie des Matériaux, Faculté des Sciences de Bizerte, 7021 Zarzouna, Tunisie; b Department of Chemical and Pharmaceutical Sciences, Centre for Structural Diffractometry, University of Ferrara, Via L. Borsari 46, I-44121 Ferrara, Italy; c Laboratoire de Chimie Organométallique de Surface (LCOMS), Ecole Superiéure de Chimie Physique Electronique, Villeurbanne Cedex, France

## Abstract

In the title compound, C_5_H_9_N_4_^+^·NO_3_^−^, the organic cations and the nitrate anions have both crystallographically imposed mirror symmetry and are linked *via* N—H⋯O hydrogen bonds, forming infinite chains running along the *c*-axis direction. The values of the N—O bond lengths [1.2256 (19)–1.2642 (18) Å] and O—N—O angles [118.39 (16)–121.64 (15)°] indicate that the nitrate anion exhibits a slightly distorted C_3*h*_ geometry. The N atom of the NH_2_ group has *sp* ^2^ character.

## Related literature

For general background to hybrid materials, see: Benali-Cherif *et al.* (2007 ▶); Messai *et al.* (2009 ▶). For studies of amine salts, see: Jayaraman *et al.* (2002 ▶); Steiner (2002 ▶). For related structures, see: Gilli *et al.* (1994 ▶); Boenigk & Mootz (1988 ▶); Jin *et al.* (2001 ▶).
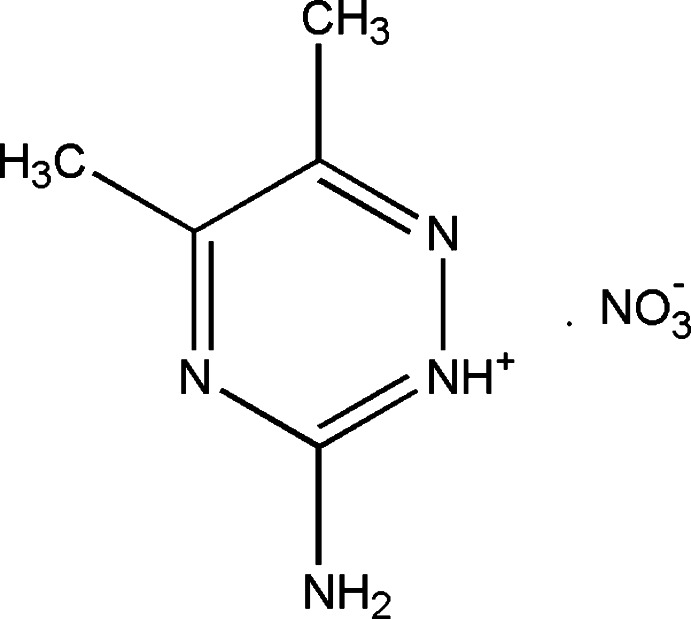


## Experimental

### 

#### Crystal data

C_5_H_9_N_4_^+^·NO_3_^−^
*M* *_r_* = 187.17
Orthorhombic, 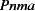

*a* = 19.7213 (2) Å
*b* = 6.4245 (2) Å
*c* = 6.7197 (6) Å
*V* = 851.38 (8) Å^3^
*Z* = 4
Mo *K*α radiation
μ = 0.12 mm^−1^
*T* = 295 K
0.32 × 0.18 × 0.12 mm

#### Data collection

Nonius KappaCCD diffractometer
2321 measured reflections
1326 independent reflections
1031 reflections with *I* > 2σ(*I*)
*R*_int_ = 0.014

#### Refinement

*R*[*F* ^2^ > 2σ(*F* ^2^)] = 0.049
*wR*(*F* ^2^) = 0.161
*S* = 1.04
1326 reflections
102 parameters
All H-atom parameters refined
Δρ_max_ = 0.24 e Å^−3^
Δρ_min_ = −0.23 e Å^−3^

### 

Data collection: *KappaCCD Server Software* (Nonius, 1997 ▶); cell refinement: *DENZO-SMN* (Otwinowski & Minor, 1997 ▶); data reduction: *DENZO-SMN*; program(s) used to solve structure: *SIR97* (Altomare *et al.*, 1999 ▶); program(s) used to refine structure: *SHELXL97* (Sheldrick, 2008 ▶); molecular graphics: *ORTEPIII* (Burnett & Johnson, 1996 ▶); software used to prepare material for publication: *SHELXL97* and *WinGX* (Farrugia, 2012 ▶).

## Supplementary Material

Crystal structure: contains datablock(s) global, I. DOI: 10.1107/S1600536813012026/rz5062sup1.cif

Structure factors: contains datablock(s) I. DOI: 10.1107/S1600536813012026/rz5062Isup2.hkl

Supplementary material file. DOI: 10.1107/S1600536813012026/rz5062Isup3.cml

Additional supplementary materials:  crystallographic information; 3D view; checkCIF report

## Figures and Tables

**Table 1:** Hydrogen-bond geometry (Å, °)

*D*—H⋯*A*	*D*—H	H⋯*A*	*D*⋯*A*	*D*—H⋯*A*
N3—H3*N*⋯O1	0.98 (4)	1.79 (4)	2.770 (2)	178 (3)
N4—H1*N*⋯O2	0.97 (3)	1.93 (3)	2.898 (2)	166 (3)
N4—H2*N*⋯O1^i^	0.87 (4)	2.18 (4)	3.043 (2)	173 (3)

Symmetry code: (i) 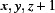.

**Fig. 1.**
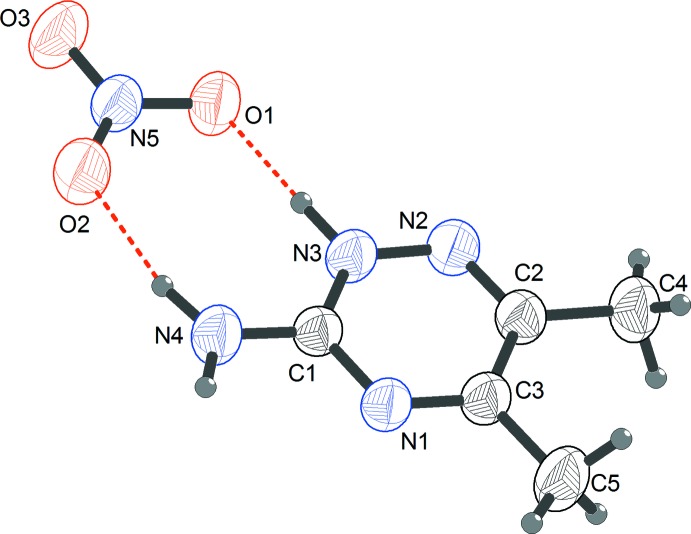
A view of the title compound, showing 50% probability displacement ellipsoids and arbitrary spheres for the H atoms. Interionic hydrogen bonds are shown as dashed lines.

**Fig. 2.**
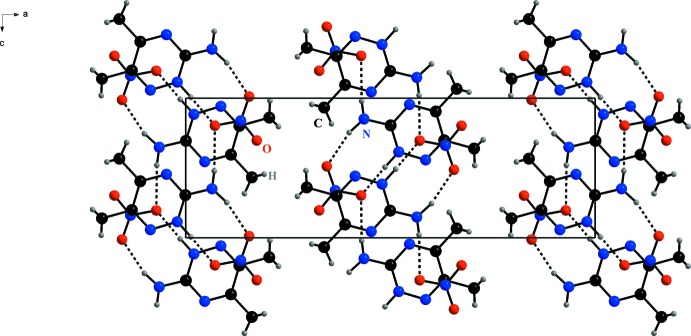
Crystal packing of the title compound, viewed down the b axis, showing the chains formed between the nitrate anions and the organic cations. Hydrogen bonds are shown as dotted lines.

**Fig. 3.**
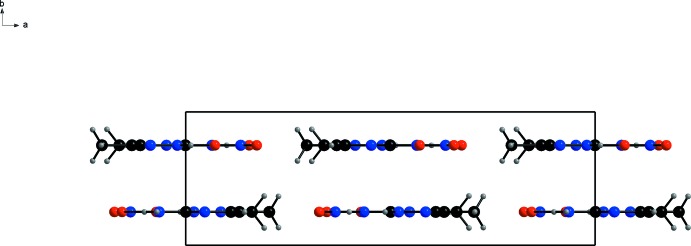
Crystal packing of the title compound viewed along the c axis.

## supplementary crystallographic information

### Comment

The blending of the organic and inorganic components in the hybrid materials allows to the development of compounds having novel properties (Benali-Cherif *et al.*, 2007; Messai *et al.*, 2009). Among these materials, salts of amines attracted more attention due to their potential importance (Jayaraman *et al.*, 2002; Steiner *et al.*, 2002). As a contribution to the study of this compound family, we report in this work the synthesis and the crystal structure of a new organic cation nitrate C_5_H_9_N_4_^+^.NO_3_^-^ (I).

The molecular structure of the title compound, as well as the atomic numbering scheme employed, are illustrated in Fig. 1. One 3-amino-5,6-dimethyl-1,2,4-triazinium cation and one discrete nitrate anion, both having crystallographically imposed mirror symmetry, comprise the asymmetric unit. The protonated N3 triazine nitrogen atom is involved in a positive charge assisted N—H···O hydrogen bond (N···O = 2.770 (2) Å) with a neighboring NO_3_^-^ anion (Gilli *et al.*, 1994). The NH_2_ unit of the cation cooperates in two N—H···O hydrogen bonds (N···O = 2.898 (2) and 3.043 (2) Å) with two neighboring nitrate anions. These hydrogen bonds link the organic entities and the nitrate anions to form infinite chains running along the *c*-axis direction (Table 1; Fig.2) and situated at *x* = n/2 and *y* ~ *n* +/- 1/4. Examination of the organic cation geometry shows that the N4–C1 distance of 1.318 (2) Å, which is of the same order of magnitude than the N–C bond of the triazine ring, clearly indicates that the N4 atom has a *sp*^2^ character. This is well confirmed by the sum of the angles around N4 equal to 360.0 (2)°. The value of the C1—N3—N2 angle [123.48 (13)°] is larger than that of the C2—N2—N3 angle [116.81 (14) °], which is consistent with the protonation of the N3 nitrogen atom (Boenigk & Mootz, 1988; Jin *et al.*, 2001). No π···π stacking interactions between the organic rings or C—H···π interactions towards them are observed. The geometrical parameters of the nitrate anion are in the normal range. The N—O bond lenghts range from 1.2256 (19) to 1.2642 (18) Å and the O—N—O bond angles range from 118.39 (16) to 121.64 (15) °], showing that the nitrate anion exhibits a slightly distorted C_3h_ geometry. These geometrical features are comparable to those previously reported for the 4-aminopyridinium nitrate salt where the N—O bond distances are in the range 1.2383 (15)–1.2632 (15) Å and the values of the O—N—O bond angles are between 118.42 (12) and 121.80 (13)°. It is worth noting that the N—O distances involving atoms O1 and O2 are longer than the third, involving atom O3. This is probably due to the fact that the later oxygen atom is not acting as acceptor of hydrogen bonding.

### Experimental

Commercial 3-amino-5,6-dimethyl-1,2,4-triazine (3 mmol) was dissolved in water/nitric acid (50:1 *v*/*v*). The resultant mixture was evaporated at room temperature. Crystals of the title compound, which remained stable under normal conditions of temperature and humidity, were isolated after several days and subjected to X-ray diffraction analysis (yield 58%).

### Refinement

Refinement was performed on F^2^ by full-matrix least-squares methods with all non-hydrogen atoms anisotropic. All H atoms were found in a difference Fourier map and refined isotropically.

### Crystal data

**Table:**

C_5_H_9_N_4_^+^·NO_3_^−^	*F*(000) = 392
*M**_r_* = 187.17	*D*_x_ = 1.460 Mg m^−^^3^
Orthorhombic, *P**n**m**a*	Mo *K*α radiation, λ = 0.71073 Å
Hall symbol: -P 2ac 2n	Cell parameters from 2321 reflections
*a* = 19.7213 (2) Å	θ = 2.0–30.0°
*b* = 6.4245 (2) Å	µ = 0.12 mm^−^^1^
*c* = 6.7197 (6) Å	*T* = 295 K
*V* = 851.38 (8) Å^3^	Rod, colourless
*Z* = 4	0.32 × 0.18 × 0.12 mm

### Data collection

**Table:**

Nonius KappaCCD diffractometer	1031 reflections with *I* > 2σ(*I*)
Radiation source: fine-focus sealed tube	*R*_int_ = 0.014
Graphite monochromator	θ_max_ = 30.0°, θ_min_ = 3.8°
φ scans and ω scans	*h* = −27→27
2321 measured reflections	*k* = −8→8
1326 independent reflections	*l* = −9→9

### Refinement

**Table:**

Refinement on *F*^2^	Primary atom site location: structure-invariant direct methods
Least-squares matrix: full	Secondary atom site location: difference Fourier map
*R*[*F*^2^ > 2σ(*F*^2^)] = 0.049	Hydrogen site location: difference Fourier map
*wR*(*F*^2^) = 0.161	All H-atom parameters refined
*S* = 1.04	*w* = 1/[σ^2^(*F*_o_^2^) + (0.1038*P*)^2^ + 0.061*P*] where *P* = (*F*_o_^2^ + 2*F*_c_^2^)/3
1326 reflections	(Δ/σ)_max_ < 0.001
102 parameters	Δρ_max_ = 0.24 e Å^−^^3^
0 restraints	Δρ_min_ = −0.23 e Å^−^^3^

### Special details

**Table:**

Geometry. All e.s.d.'s (except the e.s.d. in the dihedral angle between two l.s. planes) are estimated using the full covariance matrix. The cell e.s.d.'s are taken into account individually in the estimation of e.s.d.'s in distances, angles and torsion angles; correlations between e.s.d.'s in cell parameters are only used when they are defined by crystal symmetry. An approximate (isotropic) treatment of cell e.s.d.'s is used for estimating e.s.d.'s involving l.s. planes.
Refinement. Refinement of *F*^2^ against ALL reflections. The weighted *R*-factor *wR* and goodness of fit *S* are based on *F*^2^, conventional *R*-factors *R* are based on *F*, with *F* set to zero for negative *F*^2^. The threshold expression of *F*^2^ > σ(*F*^2^) is used only for calculating *R*-factors(gt) *etc*. and is not relevant to the choice of reflections for refinement. *R*-factors based on *F*^2^ are statistically about twice as large as those based on *F*, and *R*- factors based on ALL data will be even larger.

### Fractional atomic coordinates and isotropic or equivalent isotropic displacement parameters (Å^2^)

**Table:**

	*x*	*y*	*z*	*U*_iso_*/*U*_eq_	
O1	−0.07155 (6)	0.2500	−0.1964 (2)	0.0634 (4)	
O2	−0.15520 (7)	0.2500	0.0121 (2)	0.0705 (5)	
O3	−0.17374 (7)	0.2500	−0.3030 (2)	0.0767 (5)	
N1	0.04674 (7)	0.2500	0.4549 (2)	0.0477 (4)	
N2	0.08600 (8)	0.2500	0.0580 (2)	0.0520 (4)	
N3	0.02034 (7)	0.2500	0.11531 (19)	0.0478 (4)	
N4	−0.06438 (7)	0.2500	0.3511 (2)	0.0562 (4)	
N5	−0.13454 (7)	0.2500	−0.1615 (2)	0.0519 (4)	
C1	0.00063 (7)	0.2500	0.3055 (2)	0.0419 (4)	
C2	0.13126 (8)	0.2500	0.1986 (2)	0.0479 (4)	
C3	0.11066 (8)	0.2500	0.4046 (2)	0.0462 (4)	
C4	0.20428 (9)	0.2500	0.1392 (4)	0.0650 (5)	
C5	0.16177 (11)	0.2500	0.5668 (3)	0.0695 (6)	
H1N	−0.1005 (17)	0.2500	0.254 (5)	0.100 (9)*	
H2N	−0.0701 (14)	0.2500	0.479 (5)	0.083 (8)*	
H3N	−0.0130 (17)	0.2500	0.007 (5)	0.086 (7)*	
H1	0.2269 (9)	0.133 (3)	0.203 (3)	0.092 (6)*	
H2	0.206 (3)	0.2500	0.019 (9)	0.153 (16)*	
H3	0.1438 (16)	0.2500	0.683 (5)	0.102 (10)*	
H4	0.1920 (13)	0.130 (3)	0.544 (4)	0.128 (8)*	

### Atomic displacement parameters (Å^2^)

**Table:**

	*U*^11^	*U*^22^	*U*^33^	*U*^12^	*U*^13^	*U*^23^
O1	0.0410 (6)	0.1020 (10)	0.0472 (7)	0.000	0.0003 (5)	0.000
O2	0.0505 (7)	0.1173 (12)	0.0438 (7)	0.000	0.0051 (6)	0.000
O3	0.0532 (8)	0.1263 (14)	0.0506 (8)	0.000	−0.0160 (6)	0.000
N1	0.0417 (7)	0.0647 (8)	0.0366 (6)	0.000	−0.0006 (5)	0.000
N2	0.0452 (7)	0.0729 (9)	0.0379 (7)	0.000	0.0026 (5)	0.000
N3	0.0419 (7)	0.0646 (8)	0.0369 (6)	0.000	−0.0028 (5)	0.000
N4	0.0405 (7)	0.0801 (10)	0.0480 (8)	0.000	0.0019 (6)	0.000
N5	0.0421 (7)	0.0695 (9)	0.0441 (7)	0.000	−0.0033 (5)	0.000
C1	0.0400 (7)	0.0475 (7)	0.0381 (7)	0.000	−0.0010 (6)	0.000
C2	0.0421 (7)	0.0592 (9)	0.0424 (8)	0.000	0.0024 (6)	0.000
C3	0.0412 (7)	0.0583 (8)	0.0390 (7)	0.000	−0.0015 (6)	0.000
C4	0.0416 (8)	0.0970 (15)	0.0564 (11)	0.000	0.0075 (8)	0.000
C5	0.0474 (9)	0.1150 (18)	0.0461 (10)	0.000	−0.0072 (8)	0.000

### Geometric parameters (Å, º)

**Table:**

O1—N5	1.2642 (18)	N4—H1N	0.97 (3)
O2—N5	1.236 (2)	N4—H2N	0.87 (4)
O3—N5	1.2256 (19)	C2—C3	1.443 (2)
N1—C3	1.305 (2)	C2—C4	1.494 (2)
N1—C1	1.3547 (19)	C3—C5	1.485 (2)
N2—C2	1.300 (2)	C4—H1	0.972 (17)
N2—N3	1.3510 (19)	C4—H2	0.81 (6)
N3—C1	1.3357 (19)	C5—H3	0.86 (3)
N3—H3N	0.98 (4)	C5—H4	0.99 (2)
N4—C1	1.3183 (19)		
			
C3—N1—C1	117.14 (14)	N3—C1—N1	120.93 (14)
C2—N2—N3	116.81 (14)	N2—C2—C3	120.27 (15)
C1—N3—N2	123.48 (13)	N2—C2—C4	117.90 (16)
C1—N3—H3N	121 (2)	C3—C2—C4	121.83 (15)
N2—N3—H3N	116 (2)	N1—C3—C2	121.37 (14)
C1—N4—H1N	124 (2)	N1—C3—C5	117.75 (16)
C1—N4—H2N	111.0 (18)	C2—C3—C5	120.88 (15)
H1N—N4—H2N	125 (2)	C2—C4—H1	108.9 (11)
O3—N5—O2	121.64 (15)	C2—C4—H2	108 (4)
O3—N5—O1	118.39 (16)	H1—C4—H2	115 (2)
O2—N5—O1	119.97 (15)	C3—C5—H3	113 (2)
N4—C1—N3	120.37 (14)	C3—C5—H4	107.1 (15)
N4—C1—N1	118.70 (15)	H3—C5—H4	113.1 (17)

### Hydrogen-bond geometry (Å, º)

**Table:**

*D*—H···*A*	*D*—H	H···*A*	*D*···*A*	*D*—H···*A*
N3—H3*N*···O1	0.98 (4)	1.79 (4)	2.770 (2)	178 (3)
N4—H1*N*···O2	0.97 (3)	1.93 (3)	2.898 (2)	166 (3)
N4—H2*N*···O1^i^	0.87 (4)	2.18 (4)	3.043 (2)	173 (3)

Symmetry code: (i) *x*, *y*, *z*+1.
